# The Array of Antibacterial Action of Protocatechuic Acid Ethyl Ester and Erythromycin on Staphylococcal Strains

**DOI:** 10.3390/antibiotics11070848

**Published:** 2022-06-24

**Authors:** Maria Miklasińska-Majdanik, Małgorzata Kępa, Monika Kulczak, Maciej Ochwat, Tomasz J. Wąsik

**Affiliations:** Department of Microbiology and Virology, Faculty of Pharmaceutical Sciences in Sosnowiec, Medical University of Silesia in Katowice, Jagiellońska 4, 41-200 Sosnowiec, Poland; mkepa@sum.edu.pl (M.K.); monikakulczak7@gmail.com (M.K.); maciekochwat@wp.pl (M.O.); twasik@sum.edu.pl (T.J.W.)

**Keywords:** protocatechuic acid ethyl ester, erythromycin, fractional inhibitory concentration, *Staphylococcus* spp.

## Abstract

The spread of antibiotic resistance among bacteria has become one of the major health problems worldwide. Methicillin-resistant staphylococcal strains are especially dangerous because they are often resistant to other antibiotics. The increasing insensitivity to macrolides, lincosamides and streptogramin B antibiotics of methicillin-resistant staphylococcal isolates has limited the use of these drugs in therapy. The combination of natural compounds and antibiotics can be considered as an alternative tool to fight multi-drug-resistant pathogen infections. The aim of the presented study was to examine the antibacterial activity of protocatechuic acid ethyl ester–erythromycin combination towards *Staphylococcus aureus* and *Staphylococcus epidermidis* strains with various resistance profiles to methicillin and macrolides, lincosamides and streptogramin B (MLS_B_) antibiotics. The in-vitro antibacterial potential of the above combination was investigated by minimum inhibitory concentration assays and checkerboard testing. The observed effects were strain dependent, with 8 of 12 tested staphylococcal strains showing an indifferent effect on the natural compound and erythromycin; for 2 strains, the tested combination had an additive effect, while for another 2, the effect was synergistic. Interestingly, the multi-drug-resistant strains were more sensitive to the cooperative action of the protocatechuic acid ethyl ester and the antibiotic.

## 1. Introduction

**Figure 1:**
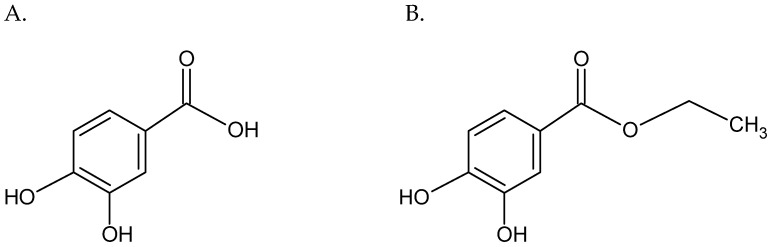
The chemical structure of protocatechuic acid (**A**) and protocatechuic acid ethyl ester (**B**).

New resistance mechanisms developed by bacteria have greatly reduced the number of available therapeutic agents effective against bacterial infections. Frequently, microorganisms isolated from hospitalized patients show resistance to more than one group of antibiotics. In recent years, multi-drug-resistant bacteria have become the major concern for global health, so the search for new antimicrobial agents is now a priority for researchers [1]. To date, the beneficial interactions of antibiotics with compounds of plant origin were suggested by many studies [2,3,4]. *Staphylococcus aureus* is one of the World Health Organization (WHO) priority pathogens for research on new antibiotics. This mainly commensal microorganism can induce diseases of the skin and soft tissues, respiratory, urinary or digestive system. What is more, it could form biofilms on medical devices or surgical sites. In turn, *Staphylococcus epidermidis,* which colonizes mucous membranes and skin, is one of the main etiological factors of nosocomial infections [2,5], and is a major element in the human body’s microbiota [6]. Colonization with coagulase-negative staphylococci (CoNS) takes place at birth and accompanies us throughout all our life [7]. It is currently believed that this opportunistic pathogen is responsible for infections associated with medical accessories, such as central venous ports, catheters, hip prostheses, knee prostheses and various procedures, such as coronary artery bypass surgery, cholecystectomy, laminectomy, colon surgery and cesarean section [8,9]. Due to the ability of *S. epidermidis* to form biofilms on the surfaces of medical devices, they are one of the reservoirs of infection [8]. Because multi-drug-resistant staphylococci strains are common in both hospital and community environments, it is very important to search for alternative treatments in staphylococcal infections. Many studies proved the synergistic effect of plant-derived compounds in combination with antibiotics [2,7,10,11,12]. New drug combinations may lead to the development of new, cheaper, more accessible, more effective, safer-for-the-environment and, above all, for patients, antimicrobial therapies [1]. Protocatechuic acid (PCA) is one of the plant-derived compounds shown to have antimicrobial activity against Gram-positive, Gram-negative bacteria and fungi [13,14,15,16]. Its antimicrobial activity is related to the ability to inhibit bacterial growth and enhance the action of antibiotics and, thus, reduce resistance development [15]. What is more, PCA is non-toxic to humans at an oral dose of 100 mg/kg [15,17]. A usage limitation of phenolic acids, such as PCA as medicinal substances, is their low bioavailability. Moreover, phenolic acids are rapidly metabolized and excreted in the urine. Chemical modifications of phenolic acids may increase their biological activity, e.g., esterification increases the lipophilicity of PCA [18,19]. Figure 1 shows the differences in the chemical structure of PCA and protocatechuic acid ethyl ester (EDHB, ethyl 3–4 dihydroxybenzoate). EDHB has not yet been extensively studied for its antibacterial activity. This compound is found in the leaves and roots of many plant species, peanut seed casings and also in tea and wine [20]. EDHB is widely used as a food stabilizer [21] and its antioxidant, neuroprotective, myo- and cardioprotective properties were indicated by many authors [21,22,23,24,25,26]. Our previous study proved the antibacterial properties of EDHB against reference and clinical strains of *S. aureus* [2]. What is more, a decrease in the minimal inhibitory concentrations (MIC) of erythromycin in the presence of this ester was observed. Therefore, it seems important to thoroughly evaluate the interactions of EDHB with erythromycin and to define its potential clinical usefulness. Since erythromycin and PCA have different targets in the bacterial cell, the choice of this combination seems to be justified. Erythromycin inhibits bacterial protein synthesis, while PCA causes membrane lysis of bacteria [16,27,28]. PCA can, thus, enhance the antibacterial effects of erythromycin without fear of interference. Since the esterification of phenolic acid may increase it bioavailability, research on the antibacterial properties of EDHB is more promising than on PCA; therefore, the authors decided to focus on EDHB. The aim of this study was to determine the direction of the influence of EDHB and erythromycin on the reference and clinical strains of *S. aureus* and *S. epidermidis*.

## 2. Results

### 2.1. Antibiotic Resistance Profile of Tested Strains

The resistance profile of the tested strains is presented in Table 1. Among the tested *S. aureus* strains, three were resistant to methicillin (*S. aureus* ATCC 43300, *S. aureus* 3 and 4) and three strains of *S. epidermidis* were resistant to this antibiotic (*S. epidermidis* ATCC 35984, *S. epidermidis* 1 and 2). The remaining isolates showed sensitivity to methicillin (*S. aureus* ATCC 25923, *S. epidermidis* ATCC 12228, *S. aureus* 1 and 2, *S. epidermidis* 3 and 4). *S. aureus* ATCC 43300, *S. epidermidis* ATCC 35984, *S. aureus* 3, *S. aureus* 4, *S. epidermidis* 1 and *S. epidermidis* 2 strains demonstrated the constitutive phenotype of resistance to MLS_B_ antibiotics (cMLS_B_). The remaining isolates did not show the MLS_B_ resistance phenotype.

### 2.2. The Fractional Inhibitory Concentration (FIC) Values for Protocatechuic Acid Ethyl Ester and Erythromycin against Staphylococcal Strains

The EDHB inhibited the growth of all the tested *S. aureus* strains, with MIC values ranging from 16 to 1024 µg/mL. The growth of the *S. aureus* reference strains was inhibited at a concentration of 512 µg/mL. *S. aureus* 1 and 2 strains proved to be sensitive to EDHB with MIC 16 µg/mL, while the growth of the *S. aureus* 3 and 4 strains was inhibited at a concentration of 1024 µg/mL. The MIC values for erythromycin against *S. aureus* isolates ranged from 0.25 to 2048 µg/mL. *S. aureus* ATCC 25925, *S. aureus* 1 and *S. aureus* 2 strains showed the lowest MIC values, 0.25 µg/mL, while *S. aureus* strains ATCC 25923, 3 and 4 demonstrated MICs at 2048 µg/mL.

EDHB at the concentrations used in this study inhibited the growth of all tested *S. epidermidis* strains. The MICs of EDHB ranged from 512 to 1024 µg/mL. Five *S. epidermidis* strains showed identical susceptibility to EDHB with MIC at 512 µg/mL. The highest MIC values at the level of 1024 µg/mL were characteristic for the *S. epidermidis* ATCC 12228 strain. The MIC values for erythromycin against *S. epidermidis* strains ranged from 0.125 to 2048 µg/mL. *S. epidermidis* ATCC 12228, 3 and 4 strains showed the lowest MIC values (0.125, 0.125 and 2 µg/mL, respectively), while *S. epidermidis* ATCC 35984, 1 and 2 strains demonstrated MICs at 2048 µg/mL. The MIC values for both EDHB and erythromycin are presented in Table 2.

Based on the checkerboard assay, MIC values were determined for EDHB in combination with erythromycin and for erythromycin in combination with EDHB.

Erythromycin and EDHB exerted an indifferent effect against five *S. aureus* strains. The synergistic effect of the compounds was noted only against *S. aureus* 3. The erythromycin–EDHB combination turned out to be more active against *S. epidermidis* strains, where in two cases, an additive effect was found (*S. epidermidis* 12228 and 3) and in one, it was synergistic (*S. epidermidis* 4). For the remaining strains, the effect of combining the compounds was indifferent.

The results of the checkerboard assay for each strain are shown in Figure S1 (Supplementary Materials), while the FIC index and their interpretation are presented in Table 3.

Table 4 shows the MIC value changes of erythromycin in the presence of EDHB at different concentrations, together with MICs of EDHB. The EDHB concentrations were determined for each strain on the basis of the MIC values obtained in the first stage of the study [29,30]. The changes in the MIC value of erythromycin after the addition of EDHB were statistically significant (*p* = 0.005). Statistical analysis also revealed significant differences between MIC changes for resistant versus susceptible strains (*p* = 0.002). Interestingly, the strains resistant to MLS_B_ antibiotics and methicillin were more sensitive to the erythromycin–EDHB combination (Table 4).

## 3. Discussion

Staphylococcal infections have become one of the most important public health problems, as multi-drug-resistant strains of this microbe are spreading rapidly. This fact stimulates scientists to search for new antimicrobial compounds and therapeutic strategies for staphylococcal disease treatment. A very promising direction of research is the implementation of substances of natural origin to augment routine antimicrobial therapies. The enhancement of antibiotic action by such compounds is due to sensitizing bacterial strains to drugs and enhancing their activity by increasing the bioavailability or simultaneously affecting a different site in the bacterial cell [4].

In this study, the antibacterial effect of the EDHB and erythromycin combination on reference and clinical *Staphylococcus* spp. strains was assessed. EDHB in combination with erythromycin showed an indifferent effect against five *S. aureus* isolates, while a synergistic interaction was found for one strain. Interestingly, a synergistic effect was noted against a multi-drug-resistant clinical strain, which, due to its character, should be less susceptible to the combined action of the compounds. In turn, among *S. epidermidis* strains, three indifferent, two additive and one synergistic effect were noted. A synergistic interaction was found against the sensitive *S. epidermidis* 4. It should be noted that if one substance is much more active, sometimes it is difficult to distinguish an indifferent effect from an additive effect, especially when using dilutions of the antibiotics [31]. The statistical analysis showed that the resistant strains of the tested staphylococci were more sensitive to the EDHB–erythromycin combination then sensitive isolates. Significant differences were observed in the decrease in erythromycin MIC values after the addition of EDHB (Table 4). Therefore, it is likely that EDHB blocks the mechanisms of bacterial resistance, thus, increasing its sensitivity.

What is more, the combination of “erythromycin–EDHB” turned out to be more effective against coagulase-negative staphylococci. Because *S. epidermidis* strains produce biofilm, which is the CoNSs’ main virulence factor, evaluation of the effective combination of EDHB–erythromycin is of importance. The antibiofilm properties of PCA have been described in many studies [32,33,34,35]. The mechanism of PCA antibiofilm action is attributed to the changes in the properties of bacterial surfaces and inhibition of quorum sensing [32,34,35]. The reference *S. epidermidis* ATCC 35983 strain has a biofilm-forming ability and possesses *icaADBC* operon. The direction of the interaction of EDHB–erythromycin for this strain was indifferent. All tested clinical *S. epidermidis* isolates also have biofilm formation ability (data not shown). Among these strains, two indifferent, one synergistic and one additive interaction were noted. Since the EDHB–erythromycin combination has been shown to be more effective against the CoNS strains, it is possible that EDHB also affects the biofilm formation process. It is believed that natural compounds showing synergism with antibiotics could have potential use in the effective treatment of nosocomial infections caused by the CoNS, especially in cases requiring a non-standard pharmacological approach [3].

Previously, Miklasińska et al. [2] investigated the antibacterial effect of EDHB alone and in combination with four antibiotics: erythromycin, clindamycin, vancomycin and cefoxitin. Twenty clinical strains and three reference strains of *S. aureus* were tested. The MIC values for EDHB ranged from 64 to 1024 µg/mL, with median 512 µg/mL, while the MIC values for EDHB in this study ranged from 16 µg/mL to 1024 µg/mL (median 512 µg/mL) and were strain dependent. In our previous study, we found that the differences in the EDHB MIC values did not depend on the mechanism of resistance to MLS_B_ antibiotics [2]. What is more, we found out that the presence of EDHB increased the sensitivity of the studied strains to erythromycin, as well as to clindamycin and vancomycin. Among examined strains were two reference isolates (*S. aureus* ATCC 43300 and *S. aureus* ATCC 25923), which were also included in this work and showed an indifferent effect to the EDHB–erythromycin combination. For the *S. aureus* ATCC 43300 strain, both studies showed the same MIC value of 512 µg/mL, while for *S. aureus* 25923, the obtained MICs were different. In our previous study, it was 256 µg/mL, while in the present work, the MIC was 512 µg/mL. As both experiments were carried out on the same strains, stored in our department and with the use of a compounds purchased from the same company, the probable cause of the difference is a laboratory error. However, it should be noted that the above MIC values do not differ significantly, and these results do not affect the assessment of the antibacterial activity of EDHB against the tested strains. The results of both studies failed to unambiguously demonstrate whether the combination of erythromycin and EDHB would bring a noticeable therapeutic benefit but showed a tendency to decrease erythromycin resistance under the influence of the EDHB.

To the best of our knowledge, there are no studies, except ours, on the antibacterial activity of EDHB, but works on the antimicrobial potential of the PCA are worth discussing. Chai et al. [36] studied the action of PCA and chlorogenic acid in combination with antibiotics against *Escherichia coli*, *S. aureus*, *Streptococcus iniae* and *Proteus mirabilis*. The authors found that the growth of Gram-negative bacteria was inhibited to a lesser extent by PCA and chlorogenic acid than the growth of Gram-positive pathogens, and that the effect of PCA was more prominent than that of chlorogenic acid. Both of these acids showed the strongest antibacterial activity against *S. aureus*. Similarly, Stojković et al. [28], in their study, noted the highest PCA activity against the *S. aureus* strain compared with other Gram-positive and also Gram-negative bacteria. The MIC value of PCA against *S. aureus* in their study was 300 µg/mL, so it was close to the median obtained in our work for EDHB (512 µg/mL). Chai et al. [36] also studied an interaction between PCA and antibiotics. A synergistic effect against *S. aureus* was noted for the PCA with clinafloxacin and gatifloxacin, an additive effect for the combination with ciprofloxacin, and an indifferent effect for the combination with sulfamonomethoxine. Sanhueza et al. [37] also assessed the interactions between PCA and antibiotics with a different mechanism of action (oxacillin, ampicillin, nalidixic acid, ciprofloxacin, norfloxacin, levofloxacin, tetracycline and chloramphenicol) against five clinical and one reference strain of *S. aureus* (ATCC 6538). The analysis of the FIC index by the checkerboard method showed values below 0.5 for all tested combinations of antibiotics, phenolic compounds and bacterial strains, which pointed to a synergistic effect. The results of the above studies may suggest that the direction of EDHB’s effect does not depend on the mechanism of action of the antibiotic. Erythromycin, used in our work, inhibits the synthesis of bacterial proteins by binding to the 50S ribosome subunit, while clinafloxacin, gatifloxacin, ciprofloxacin, nalidixic acid, norfloxacin and levofloxacin interfere with the synthesis of bacterial DNA. On the other hand, oxacillin and ampicillin interfere with the synthesis of the cell wall and tetracycline inhibits bacterial proteins synthesis by binding to the 30S ribosome subunit. Since PCA causes membrane lysis of bacteria [16,27,28], it is more probable that the synergistic effect is related to the stimulation of other sites in the bacterial cell. Because the influence of PCA–antibiotics combinations is strain dependent, it may be related to other bacterial resistance mechanisms. Mandalari et al. [38] evaluated the FIC values for catechin, PCA and epicatechin combinations against Gram-negative and Gram-positive bacteria. Further, in their study, the *S. aureus* strain turned out to be most susceptible to the examined compounds. An indifference towards synergism was observed between naringenin and PCA against *S. aureus*. Since naringenin affects the bacterial cell membrane [39], and PCA causes membrane lysis [16,27,28], the direction of the interaction may be justified by reinforcing one other’s action.

The above studies and the results of the presented work indicate that natural compounds, including EDHB, can modify the antibacterial action of antibiotics against staphylococcal strains. The experiments showed a better effect from PCA on Gram-positive bacteria, in particular, *S. aureus*, and this should be the focus of future studies. As numerous works imply the antibacterial properties of protocatechuic acid and its chemical derivatives, such studies should be carried out with a greater number of strains to precisely evaluate the antimicrobial potential of phenolic compounds, with associations with antibiotics with different mechanisms of action. Efforts should also be directed to precisely determine the mechanism of action of EDHB on bacterial cells and its potential applications in the treatment of staphylococcal infections. This line of research may, in the future, provide a new, effective method of antibacterial therapy.

## 4. Materials and Methods

### 4.1. Bacterial Strains

The antibacterial activity of EDHB was assessed against four clinical *S. aureus* strains isolated (*S. aureus* 1, 2, 3 and 4) from clinical wound samples and four clinical *S. epidermids* strains (*S. epidermidis* 1, 2, 3 and 4) isolated from blood together with four reference strains: *S. aureus* ATCC 25923, *S. aureus* ATCC 43300, *S. epidermidis* ATCC 12228 and *S. epidermidis* ATCC 35984. The species of the clinical strains was confirmed by assessing their phenotypic features, such as: morphology of colonies grown on blood agar, type of hemolysis, growth on Chapman medium and production of coagulase and catalase. The Oxoid Staphytect Plus test and the API Staph test (bioMerieux, Marcy-l’Étoile, France) were also performed. To ensure that the clinical strains were identified correctly the PCR-RFLP reaction was carried out. GeneMATRIX Tissue & Bacterial DNA Purification KIT (EuRx Ltd., Gdańsk, Poland) was used for bacterial genomic DNA isolation [40]. The PCR reaction was performed using 10× PCR RED master mix kit (BLIRT SA, Gdańsk, Poland) in a MJ Mini Personal Thermal Cycler (Bio-Rad, Hercules, CA, USA). To confirm the classification of species, the specific restriction profiles after cleaving of PCR products with 10 U of restriction enzymes XapI and Bsp143I (Fermentas, Vilnius, Lithuania) were analyzed. All the tested strains were stored in the Trypticase Soy Broth medium with 20% glycerol at −80 °C. EDHB used in this study was received from Sigma Chemical Co. (St. Louis, MO, USA) and dissolved in DMSO immediately prior to use.

### 4.2. Antibiotic Resistance Profile

The disc-diffusion method was used to assess the resistance profile of examined strains to methicillin, macrolides and lincosamides with use of antibiotic discs (EMAPOL) of cefoxitin (30 μg), clindamycin (2 μg) and erythromycin (15 μg) and Mueller–Hinton Agar (BTL) [31].

### 4.3. Susceptibility Testing of Staphylococcal Strains to Erythromycin and EDHB Using the Microdilution Method

**Figure 2:**
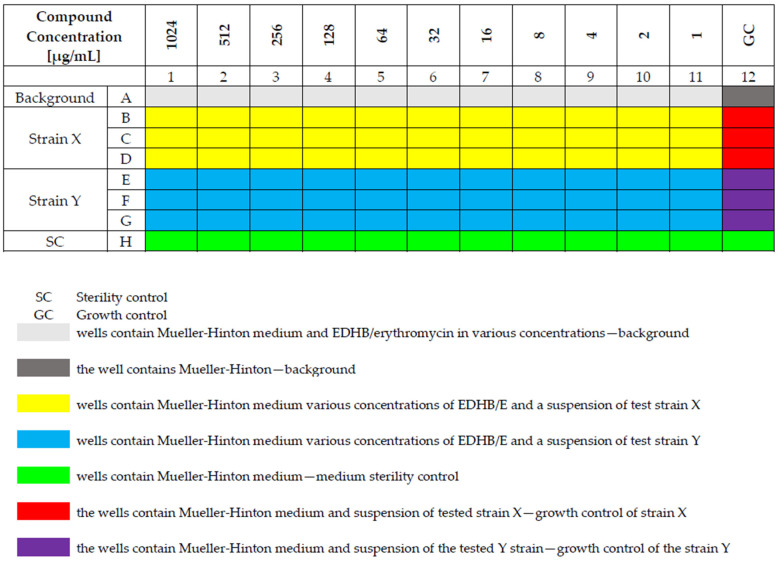
The organization of the titration plate.

The standard microdilution method in sterile 96-well polystyrene plates (FL Medical, Torreglia, Italy) was used to determine the minimum inhibitory concentrations of EDHB and erythromycin towards the staphylococcal strains [41,42]. Serial dilutions were made as follows: 11 wells of 96-well polystyrene plates were filled with Mueller–Hinton; in the next step, 100 μL of EDHB or erythromycin stock solution was added to the first well and mixed thoroughly, and subsequently 100 μL was transferred to the next and remaining wells in the same manner, and finally, from the last well 100 μL was removed. In the next step, 100 µL of the 0.5 McFarland bacterial suspension was added to the wells containing the EDHB and erythromycin dilutions. The organization of the titration plate is shown in Figure 2. The absorbance was assessed in wavelength λ = 595 nm by spectrophotometry (Thermo Electron Corp., Vantoa, Finland). The MIC is defined as the lowest compound concentration that yields no visible microorganism growth, and it indicates the resistance of bacteria to an antimicrobial agent and determines the potency of new antimicrobial agents [31,41]. All experiments were carried out in triplicate. The obtained MIC values were used to design a “checkerboard” to determine the FIC value.

### 4.4. Determination of the Susceptibility of Staphylococcal Strains to the Erythromycin and EDHB Combination

**Figure 3:**
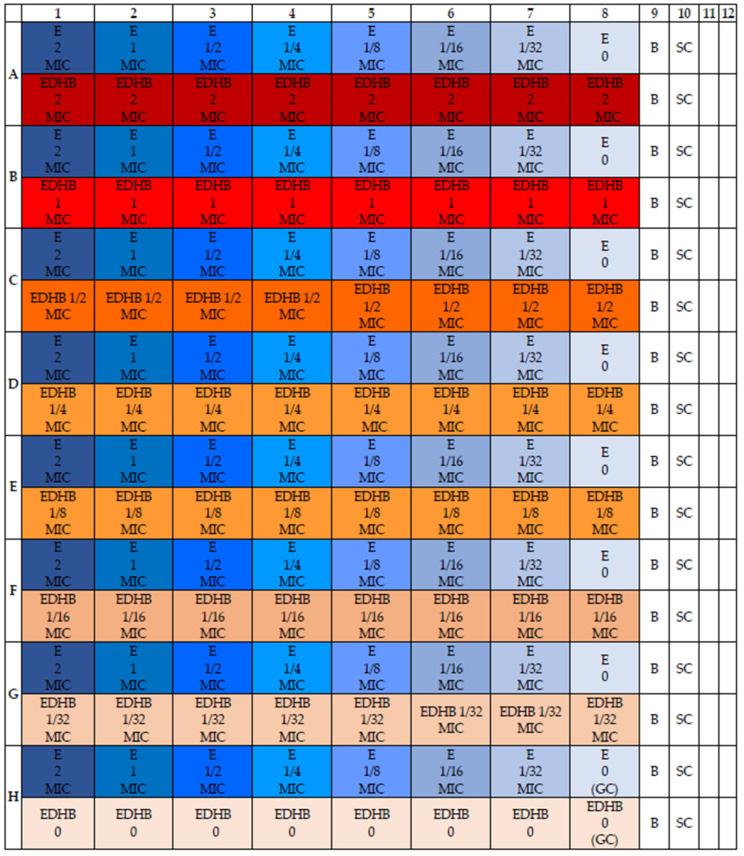
The scheme of the checkerboard assay to evaluate FIC index for tested strains. B—medium + solvent (background). SC—sterility control of the medium (sterility control). The first line of each row (A–H)—erythromycin (E) dilutions, the darkest color shows the lowest dilution (A1–H1), and the lightest shows the highest dilution (A8–H8). The second line of each row (A–H)—EDHB (protocatechuic acid ethyl ester) concentrations, the darkest color indicates the lowest dilution (A1–A8), while the lightest the highest (H1–H9).

The susceptibility of staphylococcal strains to the combination of erythromycin and EDHB was assessed by determining the fractional inhibitory concentration (FIC) value for each strain. The checkerboard microdilution method with modifications was used to determine the total susceptibility effect of the tested strains [35,36]. Briefly, the erythromycin and EDHB solutions corresponding to an MIC value of 8 were prepared. Then, a series of 1/8 MIC dilutions was made. The MIC values of the substances were determined in the previous step. As such, 95 µL of dual-concentrated Mueller–Hinton medium was added to each well of the titration plate. Then, 50 µL of EDHB and appropriate concentration of erythromycin were added. Finally, 5 µL of a 0.5 McFarland staphylococcal bacterial suspension was added. The volume of each well was 200 µL. Therefore, the solutions corresponding to the concentrations of 8 MIC, 4 MIC, 2 MIC, MIC, 1/2 MIC, 1/4 MIC and 1/8 MIC were prepared for erythromycin and EDHB to take into account the dilution of 50 µL of these substances in 200 µL of the solution and obtained as follows: 2 MIC, MIC, 1/2 MIC, 1/4 MIC, 1/8 MIC, 1/16 MIC and 1/32 MIC in each of the wells. To the last column and row, respectively, instead of EDHB and erythromycin, 50 µL of medium was added. The resulting “checkerboard” is shown in Figure 3. 

The prepared plates were incubated at 37 °C for 24 h. The absorbances were then read at 595 nm. The percent increase in individual wells in relation to the growth control was calculated using the formula:

GROWTH = (A well − A background)/(A growth control − A background) × 100%(1)

where A is the absorbance. The MIC was re-established for EDHB and erythromycin for each strain. Then the FIC [35,36] was calculated for EDHB and erythromycin and their sum for each well. The following formula was used:

FIC index = FICA + FICB = (MICA + B)/MICA + (MICB + A)/MICB(2)

where:

MICA + B—MIC of an antibiotic in the presence of a polyphenolic compound.

MICB + A—MIC of a polyphenolic compound in the presence of an antibiotic.

MICA—MIC of the antibiotic alone.

MICB—MIC of a polyphenolic compound alone.

Many different combinations are observed in the checkerboard test; therefore, only the FIC values of the most efficient combination of compounds are used to calculate the FICI [35,36]. Based on the FIC index value for each strain, the relationship between EDHB and erythromycin was assessed according to the following scale:

FIC ≤ 0.5—means synergism;

0.5 < FIC ≤ 1—means additive effect;

1 < FIC ≤ 4—means indifference;

FIC > 4—means antagonism [1].

A synergistic effect can be found when the joint effect of the substances is greater than the sum of the individual effects. An additive effect can be observed when the sum of the effects of the substances themselves is equal to the joint effect. A neutral effect is characterized by a lack of interaction between the compounds [29]. An antagonistic effect is defined as a decreased collective interaction of the compounds compared to the interaction of the compounds themselves [31].

### 4.5. Statistical Analysis

Wilcoxon signed-rank test was used to determine whether the differences in the MIC values of erythromycin after the addition of EDHB were statistically significant. To assess the relationship between the presence of resistance mechanisms and the change in erythromycin MIC values following the addition of EDHB for a given strain the t-student test was used. For all used tests *p* ≤ 0.05 was considered as statistically significant. The data were analyzed with the use of STATISTICA v 13.0 software (StatSoft, Krakow, Poland).

## 5. Conclusions

The results of this study demonstrate that multi-drug-resistant strains turned out to be more sensitive to the combination of antibiotics and EDHB than sensitive isolates. The combination of “erythromycin–EDHB” was more effective against coagulase-negative staphylococci. The in vitro additive effect and synergy of EDHB and erythromycin can indicate that EDHB is capable of augmenting the antimicrobial potential of antibiotics in vivo, but since this effect is strain dependent, further studies are necessary to evaluate the exact mechanisms of action of protocatechuic acid and EDHB.

## Figures and Tables

**Table 1:** The methicillin and MLS_B_ resistance profiles of examined strains.

Strain	Methicillin Resistance Profile	MLS_B_ Resistance Profile
*S. aureus* ATCC 25923	MSSA	-
*S. aureus* ATCC 43300	MRSA	cMLS_B_
*S. epidermidis* ATCC 12228	MSSE	-
*S. epidermidis* ATCC 35984	MRSE	cMLS_B_
*S. aureus* 1	MSSA	-
*S. aureus* 2	MSSA	-
*S. aureus* 3	MRSA	cMLS_B_
*S. aureus* 4	MRSA	cMLS_B_
*S. epidermidis* 1	MRSE	cMLS_B_
*S. epidermidis* 2	MRSE	cMLS_B_
*S. epidermidis* 3	MSSE	-
*S. epidermidis* 4	MSSE	-

MSSA—methicillin-sensitive *Staphylococcus aureus*, MRSA—methicillin-resistant *Staphylococcus aureus*, MRSE—methicillin-resistant *Staphylococcus epidermidis*, MSSE—methicillin-resistant *Staphylococcus epidermidis*, cMLS_B_—constitutive macrolide, lincosamide and streptogramin B mechanism of resistance.

**Table 2:** The MIC values for staphylococcal strains in the second stage of the study.

Staphylococcal Strain	MIC Values	
	EDHB	Erythromycin
*S. aureus* ATCC 25923	512	0.25
*S. aureus* ATCC 43300	512	2048
*S. aureus* 1	16	0.25
*S. aureus* 2	16	0.25
*S. aureus* 3	1024	2048
*S. aureus* 4	1024	2048
*S. epidermidis* ATCC 12228	1024	0.125
*S. epidermidis* ATCC 35984	512	2048
*S. epidermidis* 1	512	2048
*S. epidermidis* 2	512	2048
*S. epidermidis* 3	512	0.125
*S. epidermidis* 4	512	2

MIC—minimum inhibitory concentration, EDHB—protocatechuic acid ethyl ester.

**Table 3:** FIC index and their interpretation.

Strain	FIC Index	Interacion
*S. aureus* ATCC 25923	1.031	indifference
*S. aureus* ATCC 43300	1.016	indifference
*S. epidermidis* ATCC 12228	0.628	additive
*S. epidermidis* ATCC 35984	1.063	indifference
*S. aureus* 1	2	indifference
*S. aureus* 2	1.125	indifference
*S. aureus* 3	0.078	synergism
*S. aureus* 4	1.016	indifference
*S. epidermidis* 1	1.015	indifference
*S. epidermidis* 2	1.015	indifference
*S. epidermidis* 3	0.750	additive
*S. epidermidis* 4	0.281	synergism

FIC index—fractional inhibitory concentration index.

**Table 4:** The MIC values of erythromycin alone and in combination with EDHB towards staphylococcal strains.

Strain	MIC of EDHB	EDHB Concentration in Well with FIC Index	MIC of Erythromycin	MIC of Erythromycin with EDHB	Decrease of the MIC Value after the EDHB Addition [%]
*S. aureus* ATCC 25923	512	16	0.25	0.25	0
*S. aureus* ATCC 43300	512	512	2048	32	98.44
*S. epidermidis* ATCC 12228	1024	512	0.125	0.016	12.8
*S. epidermidis* ATCC 35984	512	32	2048	1	99.95
*S. aureus* 1	16	16	0.25	0.25	0
*S. aureus* 2	16	16	0.25	0.03125	87.5
*S. aureus* 3	1024	64	2048	32	98.44
*S. aureus* 4	1024	1024	2048	32	98.44
*S. epidermidis* 1	512	512	2048	1	99.95
*S. epidermidis* 2	512	512	2048	32	99.95
*S. epidermidis* 3	512	128	0.125	0.063	50.4
*S. epidermidis* 4	512	32	2	0.5	75

EDHB—protocatechuic acid ethyl ester, MIC—minimum inhibitory concentration.

## Data Availability

Not applicable.

## Supplementary Materials

The following supporting information can be downloaded at: https://www.mdpi.com/article/10.3390/antibiotics11070848/s1, Figure S1: The checkerboard assay results for staphylococcal strains. The dark grey represents greater than 50% bacterial growth in a well, while the light grey shows smaller than 50% bacterial growth in a well compared to the growth control. The blue represents an indifferent, pink additive, while green synergistic interaction. The red framed box represents FIC index for each strain. The values highlighted in yellow show the MICs for EDHB and erythromycin.
