# Supplementary material for: The Array of Antibacterial Action of Protocatechuic Acid Ethyl Ester and Erythromycin on Staphylococcal Strains
Source: Antibiotics (Basel). 2022 Jun 24;11(7):848. doi: 10.3390/antibiotics11070848 (PMC9311905; doi:10.3390/antibiotics11070848)
Supplement: Supplementary file 1 [file antibiotics-11-00848-s001.zip › antibiotics-1766302-supplementary.pdf]

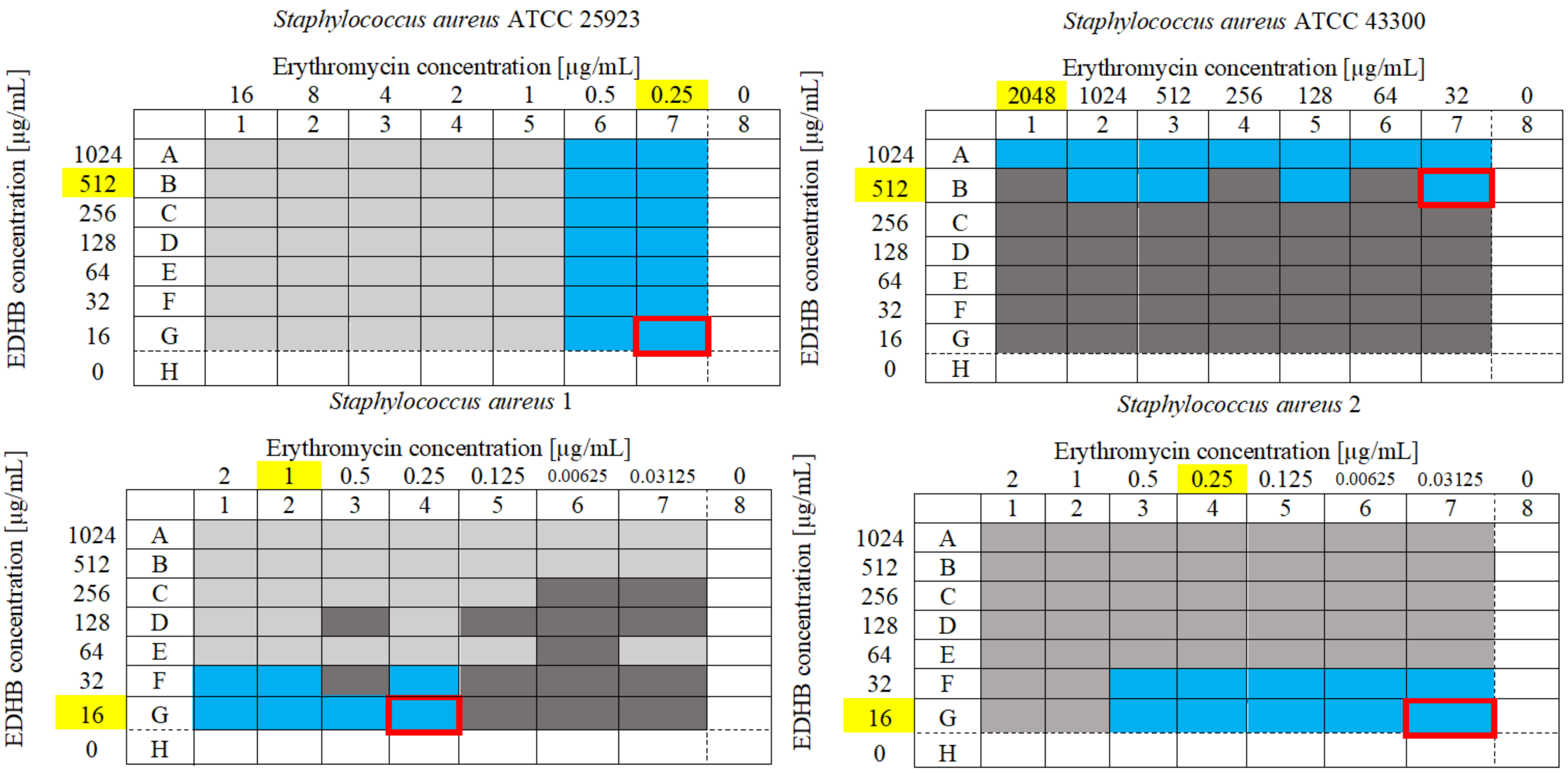

Staphylococcus aureus ATCC 25923
Erythromycin concentration [µg/mL]
16 8 4 2 1 0.5 0.25 0
1 2 3 4 5 6 7 8
EDHB concentration [µg/mL]
1024 A
512 B
256 C
128 D
64 E
32 F
16 G
0 H
Staphylococcus aureus ATCC 43300
Erythromycin concentration [µg/mL]
2048 1024 512 256 128 64 32 0
1 2 3 4 5 6 7 8
EDHB concentration [µg/mL]
1024 A
512 B
256 C
128 D
64 E
32 F
16 G
0 H
Staphylococcus aureus 1
Erythromycin concentration [µg/mL]
2 1 0.5 0.25 0.125 0.00625 0.03125 0
1 2 3 4 5 6 7 8
EDHB concentration [µg/mL]
1024 A
512 B
256 C
128 D
64 E
32 F
16 G
0 H
Staphylococcus aureus 2
Erythromycin concentration [µg/mL]
2 1 0.5 0.25 0.125 0.00625 0.03125 0
1 2 3 4 5 6 7 8
EDHB concentration [µg/mL]
1024 A
512 B
256 C
128 D
64 E
32 F
16 G
0 H

Staphylococcus aureus 3

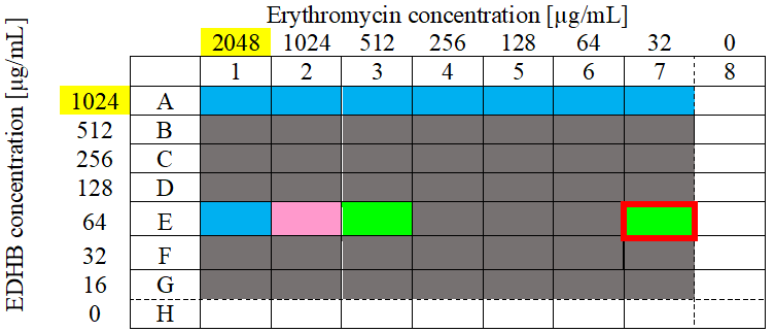

Staphylococcus aureus 4

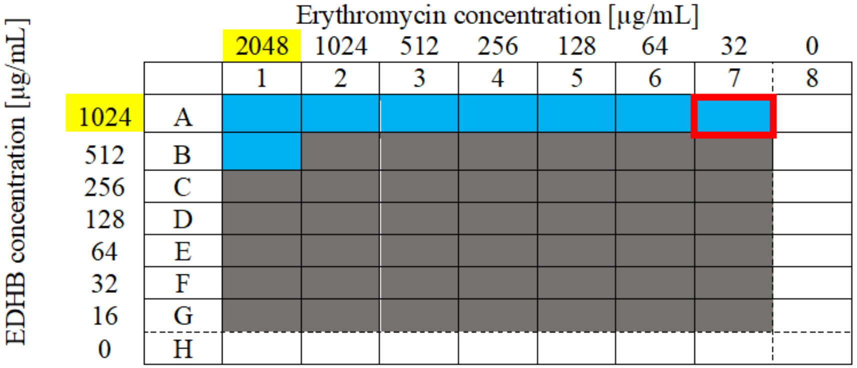

Staphylococcus epidermidis ATCC 12228

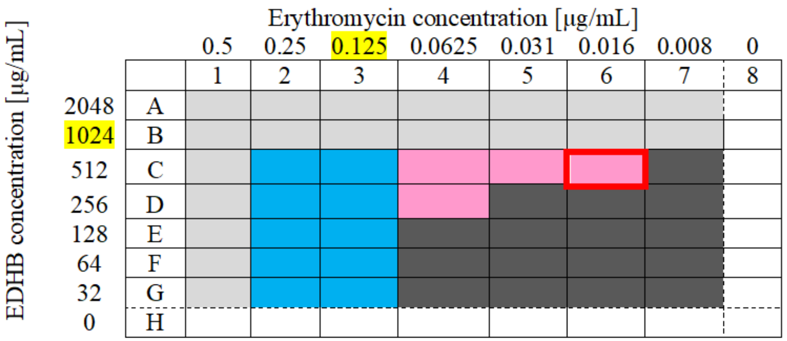

Staphylococcus epidermidis ATCC 35984

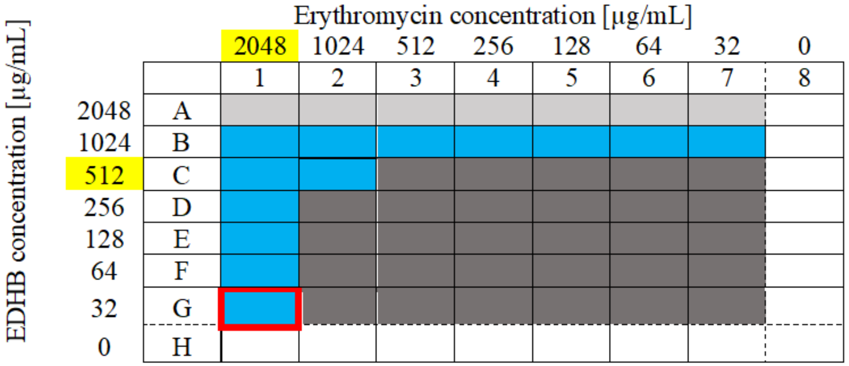

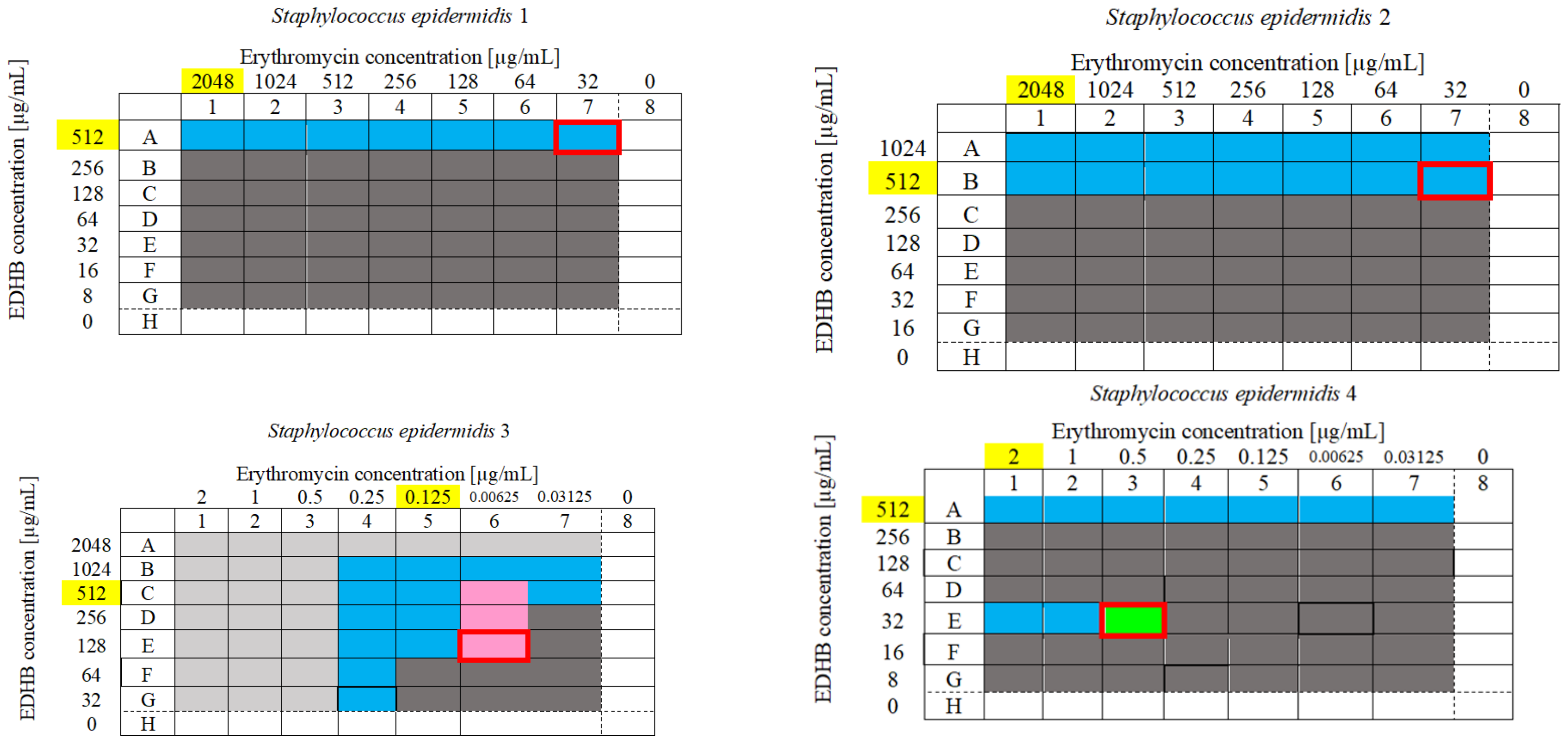

Figure S1. The checkerboard assay results for staphylococcal strains. The dark grey represents greater than 50% bacterial growth in a well, while the light grey shows smaller than 50% bacterial growth in a well compared to the growth control. The blue represents an indifferent, pink additive, while green synergistic interaction. The red framed box represents FIC index for each strain. The values highlighted in yellow show the MICs for EDHB and erythromycin.
